# Meningococcal Neonatal Purulent Conjunctivitis/Sepsis and Asymptomatic Carriage of* N. meningitidis* in Mother's Vagina and Both Parents' Nasopharynx

**DOI:** 10.1155/2017/6132857

**Published:** 2017-03-06

**Authors:** Enrique Chacon-Cruz, Jorge Arturo Alvelais-Palacios, Jaime Alfonso Rodriguez-Valencia, Erika Zoe Lopatynsky-Reyes, Maria Luisa Volker-Soberanes, Rosa Maria Rivas-Landeros

**Affiliations:** ^1^Department of Pediatrics, Hospital General de Tijuana, Tijuana, BC, Mexico; ^2^School of Medicine, Universidad Autonoma de Baja California, CISALUD Campus, Tecate, BC, Mexico; ^3^Department of Microbiology, Hospital General de Tijuana, Tijuana, BC, Mexico

## Abstract

Neonatal conjunctivitis is usually associated with vagina's infection by* Chlamydia* sp.,* N. gonorrhoeae*, and/or other bacteria during delivery. Meningococcal neonatal conjunctivitis is an extremely rare disease. We report a case of neonatal meningococcal sepsis/conjunctivitis and asymptomatic carriage of* N. meningitidis* from both parents (vagina and nasopharynx). As part of our active surveillance for meningococcal disease at the Tijuana General Hospital (TGH), Mexico, we identified a 3-day-old newborn with meningococcal conjunctivitis and sepsis. The patient had a one-day history of conjunctivitis and poor feeding. Clinical examination confirmed profuse purulent conjunctival discharge, as well as clinical signs and laboratory findings suggestive of bacteraemia. Gram stain from conjunctival exudate revealed intracellular Gram negative diplococci; we presumed the baby had gonorrheal conjunctivitis; however, serogroup Y,* N. meningitidis* was isolated both from conjunctival exudate and blood. Additionally, isolation of serogroup Y,* N. meningitidis* was obtained from mother's vagina and both parents' nasopharynx. The baby was treated with 7 days of IV ceftriaxone and discharged with no sequelae.

## 1. Case Presentation

Even though is considered to be an immediate mandatory notifiable disease, meningococcal disease (MD) is considered to be a rare condition in Mexico; however, we do believe that the current surveillance is poor and leads to poor notification of cases.

Since October 1, 2005, we have been performing active surveillance for meningococcal disease (MD) at the Tijuana General Hospital (TGH), Mexico, and have both presented and published that in the region of Tijuana, along with San Diego, California (the most transited border in the world). MD is endemic in the region, even with the presence of an outbreak during 2013 (“discussed by us, Chacon-Cruz et al. [1, 2]”).

Furthermore, we have also performed a National active surveillance among nine hospitals (“discussed by us, Chacon-Cruz et al. [3]”).

Accordingly, as part of our active surveillance for MD at TGH, we identified a newborn with meningococcal conjunctivitis and sepsis.

*N. meningitidis* was isolated by conventional culture, and serogroup identification was performed by the Pastorex meningitis kit (Alere, Ltd.®, Stockport, UK).

**Figure 1:**
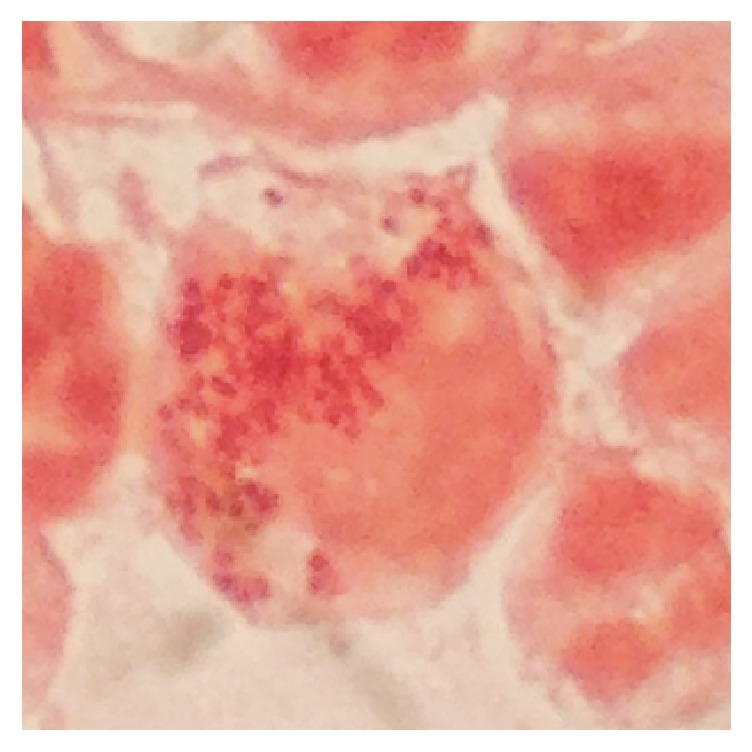
Gram stain from conjunctival exudate: abundant intracellular Gram negative diplococci.

**Figure 2:**
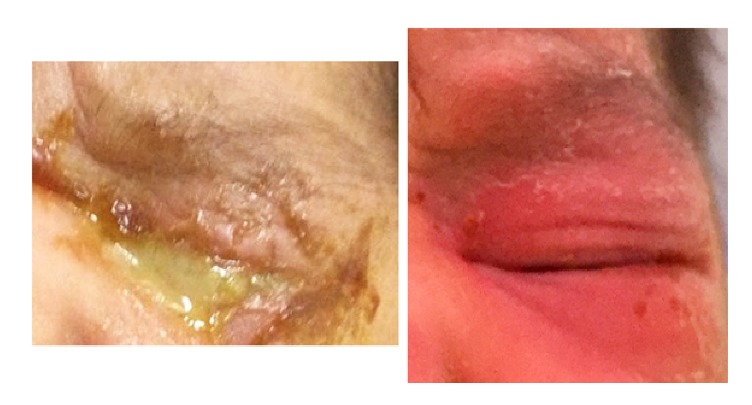
Patient's left eye before and after seven days of IV ceftriaxone.

A 3-day-old newborn delivered by vagina was admitted at TGH with a one-day history of conjunctivitis and poor feeding. Clinical examination confirmed severe blepharitis and profuse purulent discharge, as well as tachycardia (180x'), tachypnea (60x'), and irritability. CBC revealed 55,000 white blood cells (78% neutrophils), with normal hemoglobin and platelets. CSF cytochemical analysis was normal. Gram stain from conjunctival exudate revealed intracellular Gram negative diplococci (see Figure 1). We presumed the baby had gonorrheal conjunctivitis; however, serogroup Y,* N. meningitidis* was isolated both from conjunctival exudate and blood. The patient was treated with 7 days of IV ceftriaxone and discharged with resolution of his conjunctivitis and sepsis with no sequelae at the time and one month after being discharged (see Figure 2).

In addition, isolation of serogroup Y,* N. meningitidis* was obtained from mother's vagina and also from nasopharynx in both parents, who were completely asymptomatic but vaccinated with the tetravalent meningococcal conjugate vaccine (MCV4) afterwards, and also each parent received a single dose of 125 mgs of intramuscular ceftriaxone.

## 2. Discussion

Meningococcal neonatal conjunctivitis and sepsis is an extremely rare disease and apparently secondary to asymptomatic carriage from mother's vagina and/or sexual partner's nasopharynx (“discussed by De Souza and Seguro [4]”).

To date, we have also been able to find at PubMed four reports of meningococcal conjunctivitis in neonates (“discussed by Fiorito et al. [5], Kenny [6], Ellis et al. [7], Gupta et al. [8]”) among which in only one* N. meningitidis* was also found in mother's vagina (“discussed by Fiorito et al. [5]”). There is also one report of mother's vaginal colonization by* N. meningitidis* treated with amoxicillin, and further prevention of infection transmission to the newborn; in this case report,* N. meningitidis* was also present in father's nasopharynx (like in our case) (“discussed by Harriau et al. [9]”). Among these case reports, all had conjunctivitis, and one also developed meningitis (“discussed by Ellis et al. [7]”).

Our case is the first in the literature confirming the isolation of* N. meningitidis* both in conjunctival exudate and blood, as well as from mother's vagina and from nasopharynx in both parents.

The largest review of primary meningococcal conjunctivitis (PMC) comes from Barquet et al., with a review of 84 cases between 1899 and 1990. Among these patients, nine (10.7%) were newborns, and overall systemic meningococcal disease developed in 17.8%. The authors reported that development of systemic disease was significantly more frequent in patients receiving only topical therapy than in those treated with systemic antibiotics (31.71% versus 2.38%, *p* = 0.001); therefore, observation of Gram negative diplococci in a Gram stain from conjunctival exudate should be considered for systemic antibacterial therapy (“discussed by Barquet et al. [10]”).

The only limitation of our case is that we lack molecular technology to develop both Multilocus Sequence Typing (MLST) and/or Pulse Field Gel Electrophoresis (PFGE) to assure the strain isolated from the patient and both of his parents was the same; however, the fact of isolating* N. meningitidis* from the same serogroup (Y) in the same “time period” strongly suggests that the same meningococcal strain was equally present on the three subjects.

In summary, we present a very unusual case of neonatal conjunctivitis and sepsis by* N. meningitidis* serogroup Y, and acquisition of the bacteria in the newborn was highly possible by vertical transmission through mother's vagina during delivery, and the presence of this bacteria in both parents' nasopharynx suggests horizontal transmission among them.

Even though this is a very rare clinical condition,* N. meningitidis* is potentially a lethal bacteria once reaches the bloodstream; routine exudate culture (and maybe mother's vagina) from every infant with neonatal conjunctivitis should be taken, and parental antibiotics should be initiated immediately (“as discussed by Fiorito et al. [5] and Barquet et al. [10]”).
